# Knowledge and practices of Thai emergency physicians regarding the care of delirious elderly patients

**DOI:** 10.1186/s12245-014-0038-z

**Published:** 2014-09-27

**Authors:** Jiraporn Sri-on, Gregory Philip Tirrell, Prasit Wuthisuthimethawee, Shan Woo Liu

**Affiliations:** 1 Emergency Department, Vajira Hospital, Faculty of Medicine, Navamindradhiraj University, Khao street, Wachira Phayaban, Dusit 10300, Bangkok, Thailand; 2 Emergency Department, Massachusetts General Hospital (MGH), 55 Fruit St, Boston 02114, MA, USA; 3 Emergency Department, Prince of Songklanagarind University, 15 Karnchanavanich Street, Hatyai 90110, Songkla, Thailand

**Keywords:** Delirium, Elderly, Emergency department, Emergency physician, Knowledge

## Abstract

**Background:**

The Society for Academic Emergency Medicine (SAEM) Geriatric Emergency Medicine Task Force recommends assessment of delirium for all elderly emergency department (ED) patients. Little is known about emergency physicians' (EPs) opinions regarding care of delirious elderly patients. We sought to determine the knowledge and practice experience of members of the Thai Association for Emergency Medicine regarding the care of delirious elderly ED patients.

**Methods:**

We surveyed all Thai emergency physicians from July to September 2013 using a brief online survey as this does not include any non-trained physician working in the private/provincial/community EDs, still a significant part of the ED workforce in Thailand.

**Results:**

We had a response rate of 50% (239/474) of which 95% (228/239) completed the survey. Respondents largely reported that <10% of their patients experience delirium. Eighty-five percent of the respondents recognized delirium as a problem that required active intervention, and 76% of the respondents thought it was underdiagnosed in the ED. Only 24% of the respondents reported routinely screening delirium in the ED and 16% reported using a specific screening tool for delirium assessment. Forty-two percent of the respondents reported treating delirium with a long acting benzodiazepine and 29% reported using haloperidol. Forty percent of respondents thought that oversedation was the most common complication associated with drug treatment of delirium.

**Conclusions:**

Basic knowledge and perceptions surrounding the recognition, diagnosis, and treatment of delirium in elderly ED patients by Thai EPs vary. Most of the Thai EPs consider delirium in the ED an emergency condition, while far fewer screen for this condition. Future research and quality improvement should determine which single screening tool is appropriate for EPs in regular practice as well as how to standardize delirium management in the ED.

## 1 Background

Delirium, a clinical syndrome of acute decline or fluctuation in mental status and attention, is a life-threatening condition among elderly patients [1]. Delirium is associated with increased mortality, hospital length of stay, and dementia [2]-[10]. It is a condition that affects patients worldwide, regardless of income level [11].

The first opportunity to diagnose delirium is in the emergency department (ED). It occurs in 7% to 20% of elderly emergency patients [12]-[20]. The Society for Academic Emergency Medicine (SAEM) Geriatric Emergency Medicine Task Force recommends assessing for delirium in all elderly ED patients [21]. Despite this recommendation, recognition of delirium in geriatric patients by emergency physicians (EPs) is lower than 33% [12],[17]-[19]. Broad implementation of delirium assessment is dependent on the medical community's beliefs and attitudes about delirium [12],[13],[18]. In addition, most studies about the diagnosis and management of delirium have been conducted in the in-hospital setting [2]-[9]. One study in the US intensive care unit (ICU) found only 40% of health-care providers reported routinely screening for delirium and only 16% applied a validated instrument for delirium assessment [22]. While most participants believed delirium has a high prevalence and is associated with serious adverse outcomes, they reported that they had poor knowledge of its diagnosis and treatment and stated the need for better training [22],[23].

The geriatric population is increasing worldwide. Thailand is a middle-income country that has undergone an epidemiologic transition from communicable diseases to non-communicable diseases since 2002 [24]. The majority of hospitalizations in Thailand are now elderly patients [25]. The estimated prevalence of delirium on admission was 40% in one center in Thailand [26],[27]. There is insufficient data on how EPs recognize and manage delirium in Thailand. The objective of this study was to determine the knowledge and practice experience of Thai EPs regarding the care of delirious elderly patients in the ED.

## 2 Methods

### 2.1 Survey development and design

This was a descriptive cross-sectional survey design. An internet self-administered survey was sent to Thai emergency physicians and residents, who are registered in the Thai Association for Emergency Medicine (TAEM) database via email using Survey Monkey (Palo Alto, USA; http://www.surveymonkey.com). Thailand has had a formal emergency medicine residency training since 2004. The criteria for TAEM membership are the following: 1) board certified/board eligible emergency medicine, 2) attending physicians who had worked in an academic ED for more than 5 years and passed Thai ED board examination, and 3) residents in emergency medicine training. All physicians who meet the criteria have to apply for a TAEM membership. The lifelong membership rate is 1,000 Thai baths. The denominator consists of board certified/board eligible emergency medicine, EM trainees, and attending physicians who had worked in an academic ED for more than 5 years and passed Thai ED board examination. The recognition and treatment of delirium was one of the topics covered in the emergency medicine residency curriculum. Emergency medicine residents have at least one lecture about psychiatric emergencies for each study year.

The survey instrument was developed through a step-wise process that included item generation, construction, pilot testing, and clarification.

Step 1: Item generation and construction. Our survey questionnaire was adapted from Ely et al., [22] a survey given to health-care professionals working in the intensive care unit (ICU). The survey instrument consisted of a self-administered 18-item questionnaire with multiple choice, open-ended questions, and Likert scale response format. The survey contained three categories: demographics, diagnosis, and treatment experience of delirium in the ED.

Step 2: Pilot testing and clarification. The initial survey was piloted by a group of eight ED attending physicians at a tertiary, academic, and urban hospital in central Thailand. These physicians were not involved in the item generation or survey construction. Each respondent was asked to take the survey and reflect on the clarity of each survey item and the validity of each question.

Thai physicians are trained using English textbooks; hence, the survey was originally conducted in English, with some items further explained in Thai when necessary (e.g., do you routinely screen elderly patients in the ED for delirium? If yes, what tool do you use, if not, why?) We attached survey questionnaires to the manuscript as Additional file 1. We clarified five questions (question numbers 7, 12 to 14, and 16) in our survey context in Thai. The approximate time needed to fill the survey from our pilot study group was 5 min. Survey items were added or modified based on respondent feedback. This research was approved by the Institutional Review Board at the Thai hospital. Our US institutional review board exempted the requirement of informed consent.

### 2.2 Survey administration

The survey itself was distributed via email three times between July 1, 2013 and September 30, 2013. Survey responses were returned anonymously. A reminder to complete the survey was sent out via email at the end of the first and second weeks after the initial distribution of the survey, as well as via text message at the end of the third and fourth weeks after the initial distribution using a mobile messaging social application called Line (NHN Japan Corporation, Seongnam, South Korea).

### 2.3 Statistical analysis

Categorical data are presented as percentages. Chi-square and Fisher's exact tests were used to analyze categorical values where appropriate. A *p* value of ≤0.05 was considered significant.

Incomplete questions were recorded as a non-response rather than negative response in order to differentiate between completed and incomplete surveys. All incomplete surveys were excluded from analysis.

## 3 Results

**Figure 1:**
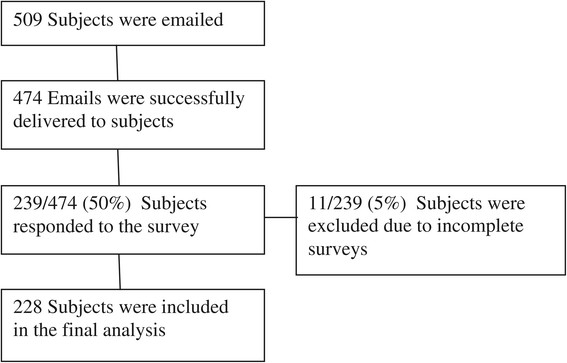
Enrollment of subjects.

Five hundred nine physicians were emailed, of which 474 emails were successfully delivered. Two hundred thirty-nine (50%) physicians responded to the survey, of which 228 completed it (95% [228/239] or 48% [228/474] of total surveys successfully sent) (Figure 1). Demographics of respondents are summarized in Table 1. Over three-fourths (77%) of the respondents were EP board certified and 117 (51%) are currently working in a university-based medical center.

**Table 1:** **Demographics of survey respondents,*n*** **= 228**

**Variable**	***n*** **(%)**
Gender	
Male	118 (52)
Type of health-care professional	
Attending physician	64 (28)
General EPs^a^	111 (49)
First year resident	17 (8)
Second year resident	10 (4)
Third year resident	26 (11)
Practice setting	
University-based medical center	117 (51)
Non-academic hospital	111 (49)
Practice experience in emergency medicine (years)	
1 to 3	72 (31)
4 to 6	89 (39)
7 to 9	54 (24)
≥10	13 (6)
Work in a hospital with annual ED volume	
<5,000	8 (3)
5,000 to 20,000	26 (11)
20,000 to 50,000	53 (23)
50,000 to 100,000	72 (32)
>100,000	24 (11)
Unsure	45 (20)

^a^General EPs: board certified emergency physicians who work in the emergency department of a hospital not affiliated with a university.

### 3.1 Incident/prevalence estimation of delirium

Out of the 228 completed surveys, most respondents reported that they either were unsure or that >40% of their ED patients are aged greater than 65 years (92 [43%] and 75 [33%], respectively). There was a wide range in estimates of the prevalence of delirium; 104 (46%) of the EPs thought that <10% of their patients experience delirium, 64 (28%) thought that <25% experience it, 15 (7%) overestimated the prevalence by estimating that ≥50% of elderly patients attending the ED experienced delirium, and 45 (19%) were unsure of the prevalence of delirious elderly patients in the ED.

### 3.2 Diagnosis of delirium

While 193 (85%) respondents agreed or strongly agreed that delirium is a problem that requires active intervention, 172 (75%) reported that delirium was underdiagnosed (Table 2).

**Table 2:** Attitudes on issues regarding delirious elderly patients in the ED

**Statement**	***n*** **(%)**				
	**Strongly agree**				**Strongly disagree**
	**5**	**4**	**3**	**2**	**1**
1. Delirium is an underdiagnosed syndrome among elderly patients.	27 (12)	145 (63)	36 (16)	20 (9)	0
2. Delirium is a problem that requires active intervention.	48 (21)	145 (64)	30 (13)	5 (2)	0
3. Delirium is largely preventable.	17 (7)	99 (43)	94 (42)	18 (8)	0
4. We overuse physical restraints on most of our elderly ED patients.	17 (7)	79 (35)	81 (36)	47 (21)	4 (2)
5. We oversedate most of our elderly ED patients.	5 (2)	42 (18)	84 (37)	90 (40)	7 (3)

### 3.3 Screening for delirium

**Figure 2:**
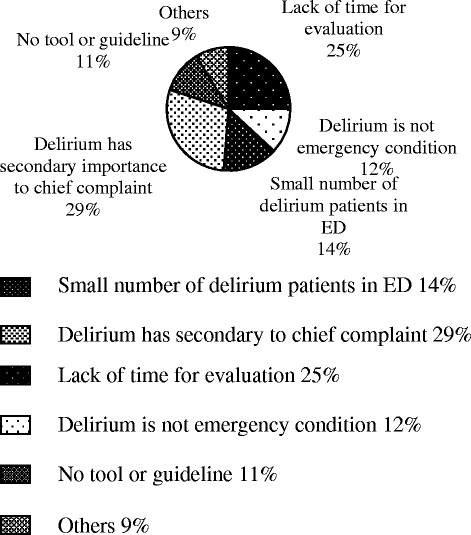
The reasons that prevent EPs from screening elderly patients for delirium.

Only 55 (24%) respondents reported routinely screening elderly patients in the ED for delirium. Screening for delirium did not significantly differ between EPs in academic (20% [23/117]) and non-academic settings (29% [32/111], *p* = 0.11). There was no statistically significant difference in delirium screening when comparing EPs with ≤6 years of experience in emergency medicine (27% [44/161]) and EPs with >6 years of experience (16% [11/56], *p* = 0.08). Also, delirium screening did not differ between the experience level of the doctor (attending physician (17% [11/64]), general EP (27% [30/111]), first-year resident (35% [6/17], second-year resident (10% [1/10]), and third-year resident (27% [7/26], *p* = 0.34). Of the physicians who did screen for delirium, the following tools were used: general clinical assessment (41 [74%]), mini-mental state examination (7 [13%]), confusion assessment method for intensive care unit (3 [5%]), delirium rating scale (2 [4%]), Glasgow coma scale (1 [2%]), and the Diagnostic and Statistical Manual of Mental Disorders fourth edition (DSM-IV) (1 [2%]). The reasons for not performing delirium screening in the ED are presented in Figure 2.

### 3.4 Treatment of delirium

Participants were asked about their pharmacological preferences for treatment of delirium and reported using a wide range of sedatives and antipsychotic drugs (Table 3). Diazepam (42%) was their pharmacologic first choice for delirium treatment, followed by haloperidol (29%), lorazepam (12%), and risperidone (5%). There was no statistical difference in the first choice of drug treatment for delirium between academic and non-academic respondents: diazepam (39% vs. 46%, *p* = 0.25), haloperidol (28% vs. 29%, *p* = 0.92) and lorazepam (12% vs.12%, *p* = 0.95). Reported adverse reactions associated with drug treatment of delirium are shown in Table 3. Oversedation (36 [41%]) was the most common reported complication, although only 47 (21%) respondents thought that EPs oversedate most elderly ED patients. More EPs (96 [42%]) thought that physical restraints were overused in most of our elderly ED patients (Table 2).

**Table 3:** Reported medication used for delirium treatment in ED and adverse reactions associated with treatment of delirium

**Variable**	***n*** **(%)**			
Medication used	First choice, (*n* = 228)	Route of administration		
		Intravenous	Intramuscular	Oral
Diazepam	96 (42)	76 (79)	0	20 (21)
Haloperidol	65 (29)	28 (43)	30 (46)	7 (11)
Lorazepam	27 (12)	7 (26)	1 (4)	19 (70)
Midazolam	13 (6)	0	13 (100)	0
Risperidone	11 (5)	0	0	11 (100)
Chlorpromazine	4 (2)	0	0	4 (100)
Olanzapine	2 (1)	0	0	2 (100)
Others	7 (3)			
Adverse reaction frequency associated with pharmacological treatment of delirium	(*n* = 89)			
Oversedation	36 (41)			
Respiratory complication	23 (26)			
Extrapyramidal side effects	14 (16)			
Hypotension	9 (10)			
Exacerbation of delirium	3 (3)			
Nausea and vomiting	2 (2)			
Death	2 (2)			

In the last 12 months, only 37 (16%) respondents had read an article related to delirium and 16 (7%) had attended a workshop or lecture relevant to delirium. There was no statistically significant difference between academic and non-academic practice setting regarding who had read an article related delirium (8% vs. 6%, *p* = 0.68) or attended a workshop relevant to delirium (15% vs.18%, *p* = 0.48).

## 4 Discussion

This study, to our knowledge, is the first reported study about emergency physicians' community perception, recognition, and treatment of delirium in the emergency room. The survey data indicated that a majority of EPs believed that delirium occasionally occurs in the ED; however, it is a serious problem and an underdiagnosed syndrome in elderly emergency patients. The incidence of this acute brain dysfunction is estimated to occur in 7% to 20% of ED patients but varies depending on patient population and screening methods used for detecting it [13],[16]-[20]. While the overall estimation of the prevalence of delirium in older ED patients was reasonably accurate, a quarter of EPs surveyed indicated that they were unsure of the prevalence of delirium or that it was overestimated.

These results are remarkably similar to those of a large survey of ICU health-care professionals in the USA [22]. In a study by Ely et al., delirium was recognized as an underdiagnosed syndrome by 78% of ICU health-care providers, with 40% routinely screening for delirium and only 16% using a delirium screening tool. In our study, respondents who did screen for delirium used tools which are not clinically validated for the evaluation of delirium in the ED (e.g., general clinical assessment, Glasgow coma scale, and mini-mental state exam). Further, 29% of the respondents believed delirium had secondary importance to the chief complaint and 12% believed delirium was not an emergency condition. Similarly, in a survey among junior doctors in UK, it was reported that only 21% had good knowledge of the diagnosis criteria for delirium [23].

Several reasons may account for why screening for delirium was reportedly low. Older patients represent an increasing population in the ED [15],[25]. They usually present with atypical signs and symptoms and many have multiple comorbidities [28]. EPs may not have been specifically trained to care for emergency geriatric patients; hence, many EPs reported being less comfortable when dealing with older patients [29]. Also, when we stratified practice experience year in ED, type of health-care professional, and practice setting, there was no difference in delirium screening reported. Education and training of EPs in the field of geriatrics should be a core competency [18],[30]. Davis and Maclullich showed physicians that had experienced in geriatric medicine were more confident in the knowledge for diagnosed delirium (28% vs.14%, *p <* 0.001) [23]. Furthermore, EPs often evaluate a large number of patients in a short period of time. When compared with many acute, life-threatening conditions, EPs normally prioritize delirium less than other complaints. Also, the infrastructure of the ED is not accommodating to the demand of an increasing number of older patients. Modification of the system may be beneficial, such as environmental changes (effective lighting, private evaluation area, noise limiting area), having social workers or nurses screening patients using validated tools to look for delirium, and having a geriatrician available to clinicians in the acute care setting [28]-[30].

Also, respondents reported that a lack of screening tools and standard guidelines for delirium limited their ability to screen elderly patients for delirium. It is possible that the respondents that reported not using a screen tool actually do use a general clinical assessment. There is no consensus in the literature regarding which delirium screening tool is the best to use in the ED. The three validated tools for delirium screening in the ED are the following: 1) the confusion assessment method (CAM), 2) confusion assessment method for the intensive care unit (CAM-ICU), and 3) a newly validated tool which combines the delirium triage screen (DTS) with the brief confusion assessment method (bCAM). The CAM takes 5 min to complete [31],[32], while the CAM-ICU takes 2 to 4 min [33], and the DTS is a <1-min assessment. DTS has only been studied at a single center and may still need to be verified as part of a multicenter study [34].

The gold standard of the treatment of delirium is identifying and treating its etiology. Non-pharmacologic strategies should be attempted first; there are only a small number of published studies supporting the safety and efficacy of pharmacologic management of delirium. In terms of pharmacologic treatment, antipsychotics and benzodiazepines are most commonly used. Older adults have an increased sensitivity to benzodiazepines and decreased metabolism of long-acting agents [35]. In general, all benzodiazepines are believed to be deliriogenic which may worsen and prolong the duration of this condition [36]-[38]. One study has shown that the receipt of benzodiazepines prior to ICU admission was associated with delirium within the first 48 h of admission [36]. Interestingly, 42.1% of the respondents to our survey reported using a long-acting benzodiazepine (diazepam) as a first line drug to treat delirium. It may have been used more frequently than short-acting benzodiazepines in our study because not all hospitals in Thailand had the intravenous form of short-acting benzodiazepines available. Haloperidol remains the most commonly used medication and is reported to be an effective agent [39]-[41]. The US Food and Drug Administration (FDA) only approved haloperidol for intramuscular injection due to a number of reports of sudden death, torsade de pointes, and QT prolongation when patients were treated with haloperidol, especially when the drug is given intravenously or at doses higher than recommended [42],[43]. Also, Thailand has the Thai FDA to oversee Thai drug safety. We follow international guidelines, especially the US FDA [44]. Thus, 43% of participants who chose haloperidol as a first line drug treatment for delirium reported using intravenous administration of haloperidol and reported a 2% (2/89) mortality rate associated pharmacological treatment of delirium. According to the pharmacological management of delirium, the respondents' knowledge might be inadequate when considering the small number of workshops attended or relevant articles read within last year, as self-reported in this study. All published evidence of the safety and efficacy of sedative and antipsychotic drugs for delirium treatment has been carried out in a non-ED hospital setting, and the ED needs more investigation into the risks and benefits of sedative and antipsychotic alternatives.

This survey had several limitations. Similar to other surveys [23],[45], the response rate was 50% and self-reporting may lead to response bias. The descriptive statistics of those who responded may systematically differ from those that did not, (for example, were younger doctors more likely to respond?) Younger doctors' level of knowledge of the treatment of delirium may significantly differ when compared to their older counterparts that did not respond to the survey. Self-reported responses were not verified by medical records, their performance in reality could be worse than reported. Many questions relied on the respondents' memory, which could be inaccurate. Also, an English questionnaire was administered to non-native English speakers which had some potential of misinterpretation. There was no question in the survey regarding the quality of EPs knowledge in English language. The assumption of EPs knowledge of English is based on Thai EPs experience preparing for board examination using English textbooks as references. Our survey results represent the knowledge and practice experience of EPs from one country, which may not be generally the same with other countries.

## 5 Conclusions

In conclusion, this survey represents a gap in basic knowledge of diagnosis and treatment of delirium in Thai EPs. Increased formal educational in geriatric emergency medicine may improve EPs recognition and diagnosis of delirium. Future quality improvement work and research should include determining which single screening tool should be implemented into EPs regular practice as well as how to standardize delirium management in the ED to maximize patient outcomes.

## Abbreviations

SAEM: The Society for Academic Emergency Medicine

EPs: emergency physicians

ED: emergency department

ICU: intensive care unit

CAM: confusion assessment method

DTS: delirium triage screen

TAEM: Thai Association for Emergency Medicine

## Competing interests

The authors declare that they have no competing interests.

## Authors’ contributions

JS and SL performed the surveys and theme-generating data analysis. JS and SL contributed to the project methodology and design. JS, SL, GT, and PW wrote the manuscript. All authors read and approved the final manuscript.

## Authors’ information

JS is an attending physician in the emergency department at the Vajira Hospital, Navamindradhiraj University, Thailand and has been selected for a research fellowship in emergency medicine at the Massachusetts General Hospital (MGH) for 2012 to 2014. GT is a graduate student from Boston University School of Medicine and a Clinical Research Coordinator in the emergency department research at MGH. PW is the chief of emergency department at the Prince of Songklanagarind University and has been selected for a Disaster fellowship at BIDMC for 2012 to 2013. SL is an attending physician in the emergency department at the MGH, Harvard Medical School.

## Additional file

## Supplementary Material

Additional file 1: Questionnaire used in this study. Click here for file
