# Supplementary material for: Knowledge and practices of Thai emergency physicians regarding the care of delirious elderly patients
Source: Int J Emerg Med. 2014 Sep 27;7:38. doi: 10.1186/s12245-014-0038-z (PMC4306074; doi:10.1186/s12245-014-0038-z)
Supplement: Additional file 1: — Questionnaire used in this study. [file s12245-014-0038-z-S1.docx]

**Additional file**

**Questionnaires** Check /

1. Gender …Male …Female Age….yr

2. Practice experience in Emergency Medicine: ………… (Years)

3. Type of healthcare professional

…Attending physician … General EP …Resident 1 …Resident 2 …Resident 3

4. Practice setting Academic

…University base Medical center ...Regional Medical Center ..Provincial hospital

…Local Community Hospital …Private Hospital

5. ED annual visit (patients/year)

… < 5,000 … 5,000-20000 … 20001-50000 … 50001-100000 ... >100,000 ...unsure

6. What percentage of the patients treated in your ED are ≥ 65 years old?

…<20 % …21-40% …41-60% …61-80% …81-100% …unsure

**Delirium** is defined as an acute decline or fluctuation in mental status and attention, an either disorganized thinking or an altered level of consciousness.

7. Approximately what percentage of your emergency elderly patients experience delirium at some point in ED?

…< 10% …10-25% …26-50% …51-75% …76-100% ...unsure

8. In your opinion, to what extent delirium a problem in elderly ED patients?

…No problem …Insignificant problem …Significant problem …Serious problem

…Very serious problem …unsure

How much do you agree with the following statement?

Strongly agree Strongly disagree

9. Delirium is an-under diagnosed syndrome among elderly patients 5 4 3 2 1

10. Delirium is a problem that requires active intervention 5 4 3 2 1

11. Delirium is largely preventable 5 4 3 2 1

12. We over use physical restrain most of our elderly ED patients 5 4 3 2 1

13. We oversedate most of our elderly ED patients 5 4 3 2 1

14. Do you routinely screen elderly patient in the ED for delirium? ... Yes ... No

If yes : What tool do you use …………………………………………………………………

If no : why?.....................................................................................................................

15. In your opinion, what is the most serious complication(s) associated with delirium?

……………………………………………………………………………………………………

16. Your medication of choice in treating delirium for elderly patients is

|  | Rank up to 3 | Route | Dose |
| --- | --- | --- | --- |
| Chlorpromazine  Diazepam  Lorazepam  Midazolam  Fentanyl  Tramadol  Propofol  Haloperidol  Olanzapine  Risperidone  Trazodone  Other |  |  |  |

17. Have you seen adverse reaction associated with treatment of delirium?

.....Yes If yes, please describe…………………………………………………………………

..... No

18. In the last 12 months, have you

A. Attended a workshop/lecture related to delirium ... Yes ...No

B. Read an article pertaining to delirium ... Yes ... No

If Yes, How many?.................................................................................................
